# Prognostic implications of troponin I elevation in emergency department patients with tachyarrhythmia

**DOI:** 10.1002/clc.23175

**Published:** 2019-03-26

**Authors:** Maribel González‐Del‐Hoyo, Germán Cediel, Anna Carrasquer, Gil Bonet, Karla Vásquez‐Nuñez, Carme Boqué, Samuel Alí, Alfredo Bardají

**Affiliations:** ^1^ Cardiology Department University Hospital of Tarragona Joan XXIII, IISPV, Rovira i Virgili University Tarragona Spain; ^2^ Cardiology Department University Hospital Germans Trias Pujol Badalona Spain; ^3^ Emergency Service Department University Hospital of Tarragona Joan XXIII, IISPV, Rovira i Virgili University Tarragona Spain; ^4^ Clinical Analysis Service University Hospital of Tarragona Joan XXIII, IISPV, Rovira i Virgili University Tarragona Spain

**Keywords:** arrhythmia, cardiac troponin, emergency department

## Abstract

**Background:**

Tachyarrhythmias are very common in emergency medicine, and little is known about the long‐term prognostic implications of troponin I levels in these patients.

**Hypothesis:**

This study aimed to investigate the correlation of cardiac troponin I (cTnI) levels and long‐term prognosis in patients admitted to the emergency department (ED) with a primary diagnosis of tachyarrhythmia.

**Methods:**

A retrospective cohort study was conducted between January 2012 and December 2013, enrolling patients admitted to the ED with a primary diagnosis of tachyarrhythmia and having documented cTnI measurements. Clinical characteristics and 5‐year all‐cause mortality were analyzed.

**Results:**

Of a total of 222 subjects with a primary diagnosis of tachyarrhythmia, 73 patients had elevated levels of cTnI (32.9%). Patients with elevated cTnI levels were older and presented significantly more cardiovascular risk factors. At the 5‐year follow‐up, mortality was higher among patients with elevated cTnI levels (log‐rank test *P* < 0.001). In the multivariable Cox regression analysis, elevated cTnI was an independent predictor of all‐cause death (hazard ratio, 1.95, 95% confidence interval: 1.08‐3.50, *P* = 0.026), in addition to age and prior heart failure.

**Conclusion:**

Patients admitted to the ED with a primary diagnosis of tachyarrhythmia and high cTnI levels have higher long‐term mortality rates than patients with low cTnI levels. cTnI is thus a biomarker with predictive capacity for mortality in late follow‐up, conferring utility in the risk stratification of this population.

## 1 INTRODUCTION

Cardiac troponin I (cTnI) is a marker of myocardial injury used in establishing a diagnosis of myocardial infarction (MI). This marker is used in addition to ischemic signs and symptoms, with chest pain being one of the most common symptom.^1^ However, certain populations, including elderly, diabetic and female patients, may have atypical presentation, leading to worse prognosis. In these populations, the suspicion of acute coronary syndrome (ACS) leads to troponin testing.^2^, ^3^, ^4^, ^5^ Consequently, it is common for patients admitted to the emergency department (ED) to undergo cTnI determination, despite not presenting cardiac‐related symptoms, such as chest pain.

Likewise, cardiac arrhythmias are very common in the ED and are a reason for cTnI determination as they may occur in the case of MI and may be associated with chest pain, syncope or palpitations. Therefore, it is common to request cTnI to rule out ACS in patients with a primary diagnosis of tachyarrhythmia in the ED. Elevated cTnI levels have been described in patients with cardiac arrhythmias not related to ACS,^6^, ^7^, ^8^, ^9^ but the long‐term prognostic value of this elevation is not yet known.

The hypothesis of this study was that patients with a primary diagnosis of tachyarrhythmia and high cTnI levels would have a worse long‐term prognosis. Therefore, our study aimed to characterize the population admitted to an ED with a principal diagnosis of tachyarrhythmia in the presence or absence of high cTnI levels, to evaluate the 5‐year all‐cause mortality and to study the prognostic implications of elevated cTnI.

## 2 MATERIALS AND METHODS

### 2.1 Study population

A retrospective cohort study involving all patients admitted to the ED with a primary diagnosis of tachyarrhythmia between January 2012 and December 2013 who underwent determination of cTnI levels at the discretion of the attending physician. The principal diagnosis of tachyarrhythmia included atrial fibrillation (AF) or atrial flutter (AFL), paroxysmal supraventricular tachycardia (PSVT) and ventricular tachycardia (VT) and was confirmed by a 12‐lead electrocardiogram. We excluded patients living outside the referral area, those below 18 years of age and those with tachyarrhythmia due to acute decompensation resulting from an underlying pathology, such as heart failure (HF), respiratory infection, chronic obstructive pulmonary disease (COPD) or secondary to type 1 MI.

### 2.2 Study variables

Patients were identified using laboratory records, and their electronic clinical records were reviewed. The study encompassed the following: demographic variables (sex and age); comorbidities, and cardiovascular risk factors; clinical variables, including reason for consultation, heart rate, blood pressure (BP), oxygen saturation (Sa02), electrocardiographic findings, laboratory tests (cTnI, hemoglobin, glycemia (blood glucose concentration) and glomerular filtration rate [eGFR] estimated using the Modification of Diet in Renal Disease‐4 [MDRD‐4]); main cardiac explorations (echocardiogram and exercise stress test). The Ethics Committee of the institution approved the study with no further need for additional informed consent.

### 2.3 Troponin I

All analyses of cTnI levels were performed using the same immunoassay techniques (cTnI‐Ultra Siemens, Advia Centaur) in the same laboratory. The reference limit for the positivity of the cTnI test was >39 ng/L, corresponding to the 99th percentile of a reference control group, with analytical imprecision expressed by a coefficient of variation below 10%. The analytic performance of this assay has been previously validated.^10^ cTnI levels were determined at the time of admission and 6 hours later. We recorded the number of cTnI determinations for each patient, the maximum value and the dynamic rise/fall patterns (changes >20% in cTnI levels). Patients with evidence of a dynamic pattern were classified as acute myocardial injury or as type 2 MI, following Saaby's criteria^11^; those without evidence of a dynamic pattern were considered to have a chronic myocardial injury.

### 2.4 Events

The primary outcome of the study was all‐cause mortality at the 5‐year follow‐up. The secondary outcomes were readmission rates for HF and for MI and the combination of all‐cause mortality and readmission rates for MI or HF at the 5‐year follow‐up. The follow‐up events were obtained from patients' electronic clinical records and death registers.

### 2.5 Statistical analysis

Continuous variables are reported as median and interquartile range (IQR). Categorical variables are presented as counts and percentages. The baseline characteristics of patients were compared using the Kruskal‐Wallis test for continuous variables and Pearson's χ^2^ test for categorical variables. Proportions were compared using Fisher's exact test. Survival analysis was performed with the Kaplan‐Meier method and compared using the log‐rank test. The associations of quantitative and qualitative variables with survival were analyzed using the univariate and multivariate Cox proportional risk models. In the adjusted model for the multivariate mortality analysis, all the variables that were significant in the univariate analysis were introduced. Backward stepwise selection was used with the input *P*‐value <0.05 to determine the predictors of mortality. To assess the discrimination benefit of adding cTnI (in a continuous manner) to a clinical model, Harrell's C statistics were used; continuous net reclassification improvement (NRI) was used for reclassification prediction. The results are presented as a hazard ratio (HR) with 95% confidence intervals (CIs). Differences were considered statistically significant at *P* < 0.05. STATA 13.0 (College Station, Texas) was used for all analyses.

## 3 RESULTS

### 3.1 Baseline characteristics

**Figure 1:**
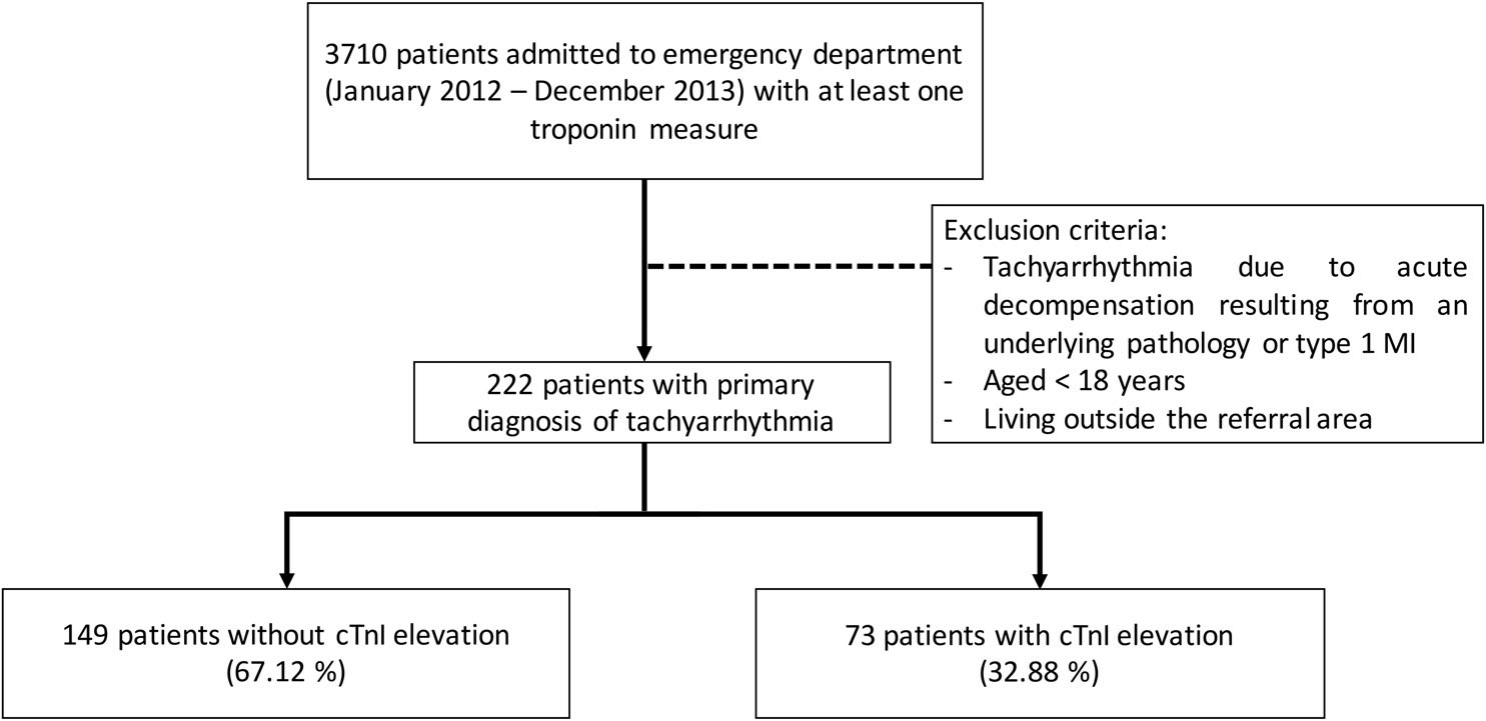
Flow diagram of patients. Distribution of patients in the two groups of the study. cTnI, cardiac troponin I; MI, myocardial infarction

A total of 1021 patients were admitted to the ED with a primary diagnosis of tachyarrhythmia between January 2012 and December 2013; of these patients, 222 patients had their cTnI levels tested (21.7%) (Figure 1). The median age was 69 years, and half were male. Of these patients, 73 (32.9%) had elevated cTnI levels. Table 1 shows the demographic, clinical and analytical variables according to the presence or absence of elevated cTnI levels. Patients with elevated cTnI levels were older, with a higher prevalence of hypertension, prior history of MI and chronic kidney disease. There were no differences in sex or in comorbidities, such as smoking, COPD, or cerebral or peripheral vascular disease, and the most frequent symptom was chest pain in both groups. At the time of admission, patients with elevated cTnI levels had higher HR (147 vs 133 bpm, *P* < 0.001), a tendency to lower SaO2, a lower eGFR (64.2 vs 84.1 mL/min/1.73 m^2^, *P* < 0.001), as well as lower values of hemoglobin (12.4 vs13.9 g/dL, *P* = 0.050) and higher glycemia (143 vs 122 mg/dL, *P* = 0.043). Of all the patients admitted, 28% of those with elevated cTnI levels needed hospital admission compared to 6% without elevated cTnI levels (*P* < 0.001). Hospital mortality was similar in both groups (*P* = 0.55). There were differences in the need for hospital admission depending on the type of tachyarrhythmia as follows: 85% of patients with VT, 12% with PSVT and 9% with AF/AFL (*P* < 0.001). The most common type of tachyarrhythmia in the total cohort was AF/AFL in 78.8% of patients, followed by PSVT in 15.3% of patients and VT in 5.9% of patients. Patients with cTnI elevation displayed higher proportions of VT compared to the group of patients without cTnI elevation (16.4% vs 0.7%, *P* < 0.001). The main diagnoses for patients with tachyarrhythmia and elevated cTnI levels according to the presence or absence of a dynamic pattern of cTnI were as follows: 29 patients (45%) had type 2 MI; 24 patients (37%) had acute myocardial injury; and 11 patients (17%) had chronic myocardial injury.

**Table 1:** Clinical baseline characteristics of patients

	Not elevated cTnI (n = 149)	Elevated cTnI (n = 73)	*P*‐value
Age, years	67 (60‐79)	73 (67‐83)	0.002
Female sex	75 (50)	38 (52.1)	0.738
Clinical history			
Diabetes	25 (16.8)	15 (20.6)	0.492
Arterial Hypertension	88 (59.1)	60 (82.2)	0.001
Current or previous smoker	38 (25.5)	20 (27.4)	0.763
Prior MI	17 (11.4)	21 (28.8)	0.001
Congestive heart failure	7 (4.7)	7 (9.6)	0.159
Peripheral arterial disease	6 (4.0)	3 (4.1)	0.977
Stroke or TIA	14 (9.4)	6 (8.2)	0.774
COPD	22 (14.8)	14 (19.2)	0.402
Renal disease	5 (3.4)	10 (13.7)	0.004
Clinical symptoms			
Chest pain	58 (38.9)	37 (50.7)	0.096
Dyspnoea	19 (12.8)	7 (9.6)	0.491
Syncope	4 (2.7)	6 (8.22)	0.062
Other	86 (57.7)	32 (43.8)	0.051
Electrocardiogram			
ST‐segment depression	4 (2.7)	5 (7.2)	0.148
Negative T wave	7 (4.8)	5 (7.2)	0.527
Vital signs on admission			
Maximum HR, bpm	133 (113‐153)	147 (133‐160)	<0.001
SBP, mmHg	130 (113‐145)	129 (115‐150)	0.928
SaO2,%	98 (96‐100)	97 (96‐99)	0.053
Laboratory tests			
Glycemia, mg/dL	122 (97‐129)	143 (103‐153)	0.009
Hemoglobin, g/dL	14 (13–15)	12 (12‐15)	0.050
eGFR, mL/min/1.73 m^2^	84 (66‐100)	64 (46‐80)	<0.001
Maximum level of cTnI, ng/L	0.01 (0.01‐0.02)	1.61 (0.08‐0.63)	<0.001
Clinical management			
Echocardiogram	13 (8.72)	25 (34.25)	<0.001
Exercise stress test	1 (0.67)	2 (2.74)	0.252

Data are presented as No. (%) or median and interquartile range (IQR).

Abbreviations: COPD, chronic obstructive pulmonary disease; cTnI, cardiac troponin I; eGFR: estimated glomerular filtration rate; HR, heart rate. SBP, systolic blood pressure; SaO2, arterial oxygen saturation; TIA, transient ischemic attack.

### 3.2 Events

**Figure 2:**
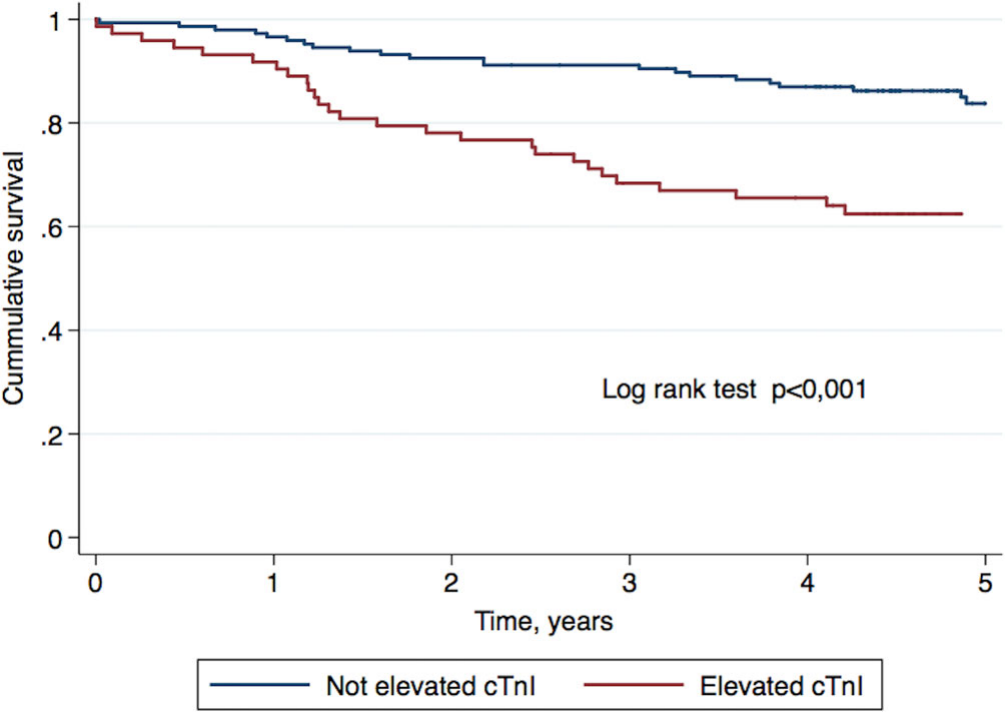
Kaplan‐Meier survival at 5‐year follow‐up in patients with and without elevated cardiac troponin I levels

During a median follow‐up of 56 months (IQR 49‐64), 51 patients died (23%), 21 patients were readmitted for HF (10%), and 5 patients were readmitted for MI (2%). Patients with elevated cTnI levels had a higher 5‐year all‐cause mortality (log‐rank test *P* < 0.001) (Figure 2), evident 2 years after diagnosis and increasing after that (Table 2). There were no differences in readmission due to HF or MI. In combined events at 5 years, patients with cTnI elevation had a worse prognosis, mainly driven by increased mortality. It is notable that within the first 30 days, there were no differences in mortality or readmissions due to HF between the two groups, and none of the patients were readmitted for MI. Elevated cTnI levels were found to be a marker associated with higher mortality at the 5‐year follow‐up in the univariate Cox regression analysis (HR: 2.67, 95% CI: 1.54‐4.63, *P* < 0.001), together with other variables, such as age, prior MI, prior HF and initial presentation with syncope, glycemia, eGFR and anemia. In the multivariate Cox regression analysis, cTnI elevation remained associated with mortality (HR: 1.95, 95%CI: 1.08‐3.50, *P* = 0.026), in addition to age and prior HF (Table 3). The addition of cTnI to a clinical model (containing variables independently associated with the primary endpoint as follows: age, prior HF, and glycaemia) improved discrimination, increasing Harrell's C statistics from 0.78 to 0.81; the associated continuous NRI was 0.43 (95%CI: 0.09‐0.84), corresponding to 15.1% of patients being reclassified.

**Table 2:** Five‐year follow‐up events in different time ranges

Event	Follow up	Not elevated cTnI (n = 149)	Elevated cTnI (n = 73)	*P‐*value
Mortality	30 days	1 (0.67)	1 (1.37)	0.605
	1 year	5 (3.36)	6 (8.22)	0.117
	2 years	11 (7.38)	16 (21.92)	0.002
	4 years	19 (12.75)	25(34.25)	<0.001
	5 years	22 (14.77)	27(36.99)	<0.001
Readmission for MI	30 days	0 (0)	0 (0)	1.000
	1 year	2 (1.34)	0 (0)	1.000
	2 years	3 (2.01)	0 (0)	0.553
	4 years	4 (2.68)	1 (1.37)	1.000
	5 years	4 (2.68)	1 (1.37)	1.000
Readmission for HF	30 days	3 (2.01)	1 (1.37)	1.000
	1 year	3 (2.01)	2 (2.74)	0.665
	2 years	4 (2.68)	5 (6.85)	0.159
	4 years	8 (5.37)	7 (9.59)	0.239
	5 years	13 (8.72)	7 (9.59)	0.833
Combined event (mortality, readmission for MI, readmission for HF)	30 days	4 (2.68)	2 (2.74)	0.643
	1 year	9 (6.04)	8 (10.96)	0.195
	2 years	16 (10.74)	19 (26.03)	0.003
	4 years	27 (18.12)	30 (41.10)	<0.001
	5 years	35 (23.49)	32 (43.84)	0.002

Abbreviations: cTnI, cardiac troponin I; HF, heart failure; MI, myocardial infarction.

**Table 3:** Univariate and multivariate predictors for 5‐year all‐cause mortality

	Univariate	*P*‐value	Multivariate	*P*‐value
	HR 95% CI		HR 95% CI	
Age	1.10 (1.06‐1.14)	<0.001	1.09(1.05‐1.13)	<0.001
Female sex	1.05 (0.61‐1.82)	0.852	—	—
MI	2.25 (1.23‐4.10)	0.008	—	—
HF	3.70 (1.73‐7.91)	0.001	2.55(1.15‐5.65)	0.021
Syncope	3.81 (1.62‐8.95)	0.002	—	—
cTnI elevation	2.67 (1.54–4.63)	<0.001	1.95 (1.08‐3.50)	0.026
Hemoglobin	0.76 (0.67‐0.87)	<0.001	—	—
eGFR(mL/min/1.73 m^2^)	0.97 (0.95‐0.98)	<0.001	—	—
Glycemia	1.01 (1.00‐1.01)	<0.001	1.00(1.00–1.01)	0.035

Abbreviations: CI, confidence interval; cTnI, cardiac troponin I; eGFR, estimated glomerular filtration rate; HF, heart failure; HR, hazard ratio; MI, myocardial infarction.

## 4 DISCUSSION

This study shows that one‐third of patients with a principal diagnosis of tachyarrhythmia admitted to the ED had elevated cTnI levels. This group of patients was identified as a high‐risk population due to older age, worse cardiovascular risk profile, and higher long‐term mortality. Therefore, cTnI emerges as a biomarker with predictive capacity for mortality in long‐term follow‐up, conferring utility in the stratification of risk in this population. In the ED, cTnI testing is a common tool used for the diagnosis of MI. However, elevated cTnI levels are frequently encountered in various clinical situations, such as pulmonary thromboembolism, HF, and sepsis and is associated with poor prognosis.^12^, ^13^, ^14^ In previous studies, it has been shown that a high proportion of patients admitted to the ED present cTnI elevation in clinical contexts other than acute atherothrombotic plaque disruption (type 1 MI) and that this elevation is associated with a worse prognosis in follow‐ups.^15^, ^16^ In addition, it has been observed that the prognosis was similar in patients who fulfill diagnostic criteria for type 2 MI and for non‐ischemic myocardial injury, being worse compared to patients with type 1 MI.^17^ Moreover, patients with cTnI elevation and without chest pain have been found to present higher mortality rates.^18^ In addition, when comparing different laboratory troponin testing in patients in the ED without ACS, cTnT, and cTnI elevations have both important prognostic information regarding all‐cause mortality.^19^ In conclusion, these data indicate that cTnI elevation is a reflection of acute or chronic myocardial injury that is not always attributable to ACS and should be considered an entity in itself.^20^

From a diagnostic point of view, it is possible that a significant proportion of our patients met the diagnostic criteria for type 2 MI or non‐ischemic myocardial injury, as described in other works.^6^, ^10^, ^21^ However, it can be difficult to differentiate between the two as they may coexist. In our study, 83% of the patients with a primary diagnosis of tachyarrhythmia and elevated cTnI showed a dynamic pattern, meeting the criteria for type 2 MI and acute myocardial injury in similar proportions. Nonetheless, during follow‐up, short‐ and long‐term prognoses were worse, regardless of the type of myocardial injury.

In patients with a primary diagnosis of tachyarrhythmia, several studies have described the presence of cTnI elevation. Chow et al^22^ retrospectively studied 78 patients who attended an ED due to PSVT. Elevated cTnI levels were observed in 37.2% of the patients, which was a proportion similar to that observed in our study, despite the population being younger and with fewer cardiovascular risk factors. In the multivariate analysis, prior coronary heart disease, HR at admission and ventricular dysfunction were predictors of cTnI elevation. At the 4‐year follow‐up, elevated cTnI levels were associated with an increased risk of the combined event of all‐cause death, MI, and cardiovascular readmission (HR: 3.67, 95% CI: 1.22‐11.1, *P* = 0.02). This result was mainly driven by cardiovascular readmissions, unlike the observations in this study. Mariathas et al^23^ observed elevated cTnI levels (Beckman Coulter Access) in 46.8% of patients in a retrospective study of patients with different types of primary tachyarrhythmia. Consistent with our results, patients with elevated cTnI levels showed a higher mortality rate compared to patients without cTnI elevation, with mortality rates similar to patients with MI and without ST‐segment elevation.

The presence of underlying coronary disease in patients with tachyarrhythmia and elevated cTnI levels has been the subject of controversy and research. In a study by Yedder et al,^24^ 73 patients with PSVT were analyzed, and 32.9% presented elevated cTnI levels. After carrying out MI detection studies in 9 patients with elevated cTnI levels and in 11 without cTnI elevation, only 1 patient had significant coronary artery disease requiring revascularization. They concluded that tachyarrhythmia is associated with elevated cTnI levels, but less than 10% of these patients show significant severe coronary disease. This finding is consistent with that of Schueler et al study.^25^ They analyzed the association between intranodal tachycardia and ultrasensitive cTnT elevation (Roche Diagnostics, Mannheim, Germany) in 139 patients, reporting elevation in 45 patients (32.3%), which is a proportion similar to this study. To rule out underlying coronary disease, non‐invasive angiography was performed in 12 patients and invasive coronary angiography in 32 patients, demonstrating significant coronary disease in 50% of patients. The authors concluded that the cTnT elevation in patients with intranodal tachycardia occurred even in the absence of coronary disease. Sayadnik et al^26^ studied 70 patients admitted to an ED with PSVT, 74% of whom had elevated levels of ultrasensitive cTnT (Elecsys 2010 Platform Roche Diagnostics, Penzberg, Germany). The patients were older in that study than in this study and presented mainly with chest pain. Those patients underwent screening for ischemic heart disease (with a nuclear stress test performed on 23 patients, coronary angiography on 8 patients and coronary computed tomography on 5 patients), which was positive in 21.7% of the patients. Comparable to our results, the authors observed that the increase in cTnT was higher and of greater duration in those with underlying coronary disease. The aforementioned studies show that underlying coronary disease is not present in a significant proportion of patients with primary tachyarrhythmia or troponin elevation, explaining only partially the elevation of the cardiac biomarker in this clinical setting.

HF patients are predisposed to develop arrhythmias. It has been described that highly complex, interactive, and dynamic changes in mechanical, structural neurohormonal properties might predispose the failing heart to tachyarrhythmias. In our study, there were no differences in the history of HF between the cTnI elevation groups or readmission for HF in the long term, but prior HF was an independent mortality risk factor in the long term. This result may reflect a relationship between tachyarrhythmias and structural changes describing an underlying structural heart disease, which might explain cTnI elevation.

Therefore, the mechanisms justifying elevated cTnI concentrations in tachyarrhythmia may be multiple and influenced by other factors that are not yet fully clear. It is possible that shortening of the diastolic period during tachyarrhythmia leads to the appearance of subendocardial ischemia, which explains the elevation of cardiac biomarkers.^27^ Another potential pathophysiological mechanism was described by Goette et al^28^ in a porcine model subjected to rapid atrial stimulation, showing that a decrease in coronary flow reserves may induce an alteration in the microcirculation of the left ventricle. In this study, several markers of oxidative stress mediated by angiotensin II were increased in pigs subjected to rapid atrial pacing. In other experimental studies, it has been observed that myocardial overdistension represents another tachycardia‐dependent mechanism that would justify the troponin elevation, since there is a direct association between the parallel increase in proBNP and troponins.^29^ It is also possible that changes in the permeability of the cardiac myocyte membrane secondary to myocardial injury may be sufficient to generate the release of cardiac troponin to the cytoplasm, without structural damage being seen.^30^ Thus, overall, it seems very likely that the cause of cTnI elevation in tachyarrhythmia is multifactorial and is more frequent in the elderly population and in patients with multiple cardiovascular risk factors and comorbidities.

Because in our study, the adverse prognosis of patients with elevated cTnI levels was determined on an all‐cause basis and not by readmission rates for cardiovascular causes, we consider that the performance of ischemia detection studies should be established individually, according to the pre‐test probability of ischemic heart disease and the clinical characteristics of the acute episode. An important potential practical implication of our observations is the expectation of elevated cTnI levels among one‐third of patients admitted to the ED with tachyarrhythmias. Thus, our findings suggest that cTnI assessment in these patients could be a useful tool for long‐term mortality risk prediction, in parallel to a clinical assessment of conventional risk factors. To date, no interventions have been described that are capable of modifying the adverse prognosis in this population. Hence, close monitoring seems to be the most reasonable therapeutic strategy.

### 4.1 Limitations

This study has certain limitations. First, cTnI testing in the ED was left to the decision of the treating physician, who may not have measured cTnI in patients with a lower risk perception, such as younger adults with fewer cardiovascular risk factors. This decision could represent a selection bias. Despite this limitation, the study reflects the real‐life clinical scenario in the ED. Second, as it was a retrospective study, the evaluation of coronary heart disease was not performed in all patients with elevated cTnI; hence, we cannot draw conclusions on this aspect. However, in the short‐ and long‐term follow‐up, we did not observe readmission for MI or HF. This result strongly suggests that elevated cTnI levels do not correlate closely with the burden of coronary atherosclerotic disease. Third, the study did not aim to identify the mechanism by which elevated cTnI levels predict cardiovascular risk in the context of tachyarrhythmia.

## 5 CONCLUSIONS

This study shows that cTnI elevation in patients with a primary diagnosis of tachyarrhythmia admitted to the ED is frequent and is associated with an increased risk of long‐term mortality. These patients presented a worse cardiovascular risk profile and more comorbidities. Our data prompt the need for more research to understand the mechanisms of elevated cTnI levels in patients with tachyarrhythmia and the association with cardiovascular risk.

## CONFLICT OF INTEREST

The authors declare no potential conflict of interests.
